# A nonparametric random coefficient approach for life expectancy growth using a hierarchical mixture likelihood model with application to regional data from North Rhine-Westphalia (Germany)

**DOI:** 10.1186/1471-2288-13-36

**Published:** 2013-03-09

**Authors:** Dankmar Böhning, Sarah Karasek, Claudia Terschüren, Rolf Annuß, Rainer Fehr

**Affiliations:** 1 Southampton Statistical Sciences Research Institute, Mathematics and Medicine, University of Southampton, Southampton SO17 1BJ, UK; 2 Institute of Statistics, Graz University of Technology, Kopernikusgasse 24/III, 8010 Graz, Austria; 3, Landeszentrum Gesundheit Nordrhein-Westfalen / NRW Centre for Health, Westerfeldstr. 35/37, 33609 Bielefeld, Germany

**Keywords:** Likelihood–based cluster analysis, Random coefficient modelling, Finite mixture model, Life expectancy

## Abstract

**Background:**

Life expectancy is of increasing prime interest for a variety of reasons. In many countries, life expectancy is growing linearly, without any indication of reaching a limit. The state of North Rhine–Westphalia (NRW) in Germany with its 54 districts is considered here where the above mentioned growth in life expectancy is occurring as well. However, there is also empirical evidence that life expectancy is not growing linearly *at the same level* for different regions.

**Methods:**

To explore this situation further a likelihood-based cluster analysis is suggested and performed. The modelling uses a nonparametric mixture approach for the latent random effect. Maximum likelihood estimates are determined by means of the EM algorithm and the number of components in the mixture model are found on the basis of the Bayesian Information Criterion. Regions are classified into the mixture components (clusters) using the maximum posterior allocation rule.

**Results:**

For the data analyzed here, 7 components are found with a spatial concentration of lower life expectancy levels in a centre of NRW, formerly an enormous conglomerate of heavy industry, still the most densely populated area with Gelsenkirchen having the lowest level of life expectancy growth for both genders. The paper offers some explanations for this fact including demographic and socio-economic sources.

**Conclusions:**

This case study shows that life expectancy growth is widely linear, but it might occur on different levels.

## Background

Life expectancy in Germany is increasing unbrokenly at linear rate. This corresponds to a world–wide trend – despite controversial statements (see also Oeppen and Vaupel [1] for this point). But life–expectancy does not increase on the same level for everyone. Regional data from North Rhine-Westphalia (NRW) are available for the 21 years from 1990 to 2010 and the 54 administrative regional districts of the state NRW. Continuous analyses of these data are an important part of the health reporting in NRW. Results show which health gains are realized and where higher efforts are needed. The paper suggests a cluster-analytic approach to identify the various components of different levels of growth in life-expectancy. Socio-spatial cluster analysis on the basis of the 54 regions has been done previously including Strohmeier *et al.*[2]. Strohmeier *et al.* could identify six clusters on the basis of social indicators for NRW, which classified the 54 districts into sub-types with interpretable labels. However, the approach by Strohmeier *et al.* did not include temporal modelling. The approach proposed here is focusing on temporal–spatial modelling of life expectancy with the aim of identifying spatial clusters in life expectancy growth, ultimately targeting on constructing a life expectancy growth map of NRW. The approach is less focussed on explaining differentials in life expectancy by other factors, say socio–economic factors (Gallo *et al.*[3]), in the sense of an ecological study analysis, although we will take up this string in the discussion.

**Figure 1:**
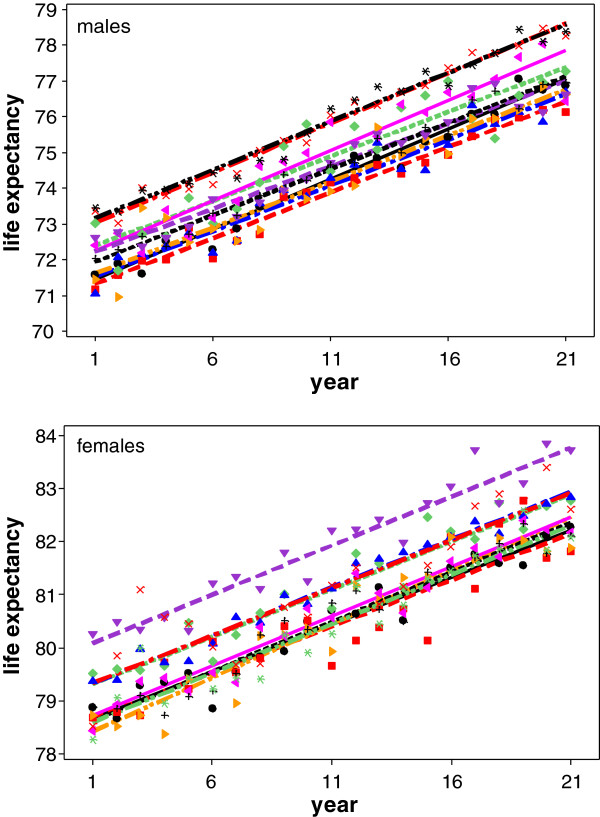
Life expectancy for men resp women for 10 randomly selected regions; the lines are regression lines fitted separately for each of the 10 regions.

In a nutshell, the approach is as follows. For each of the 54 regions a straight line model *Y*_*t*_ = *α* + *βt* + *ϵ* is assumed for the life expectancy *Y*_*t*_ at year *t*. Here, *α* and *β* are the intercept and slope of the line, respectively. As also Figure 1 suggests, we will model each of the 54 regions with identical slope but potentially different intercepts or levels of growth. The key point is that we will focus with our cluster–analysis on the variation of the different levels of growth and try to identify different components if present. The paper is organized as follows. In the next section, some background information of the life expectancy data and the region they stem from is given. In Section ‘Methods’, the nonparametric mixture model used for the cluster analysis is presented in parallel with the associated likelihoods. It discusses the EM algorithm (Dempster, Laird and Rubin [4], McLachlan and Krishnan [5], McLachlan and Peel [6]) for finding maximum likelihood estimates as well as classifying the regions into the mixture components (clusters). Section ‘Results’ presents the results of the analysis including maps of the estimated cluster structure. The paper closes with a discussion which tries to put the findings into perspective.

### Data

NRW is the most populous state of Germany, with four of the country’s ten largest cities. Its capital is Düsseldorf. The state consists of five provinces (Regierungsbezirke), until 2010 divided into 31 rural districts (Kreise) and 23 urban districts (kreisfreie Städte), forming the above mentioned total of 54 districts which is the basis of our analysis. The underlying dataset ‘LifeexpectancyNRW.xls’ consists of two sheets, separately aggregated according to gender, each with *N* = 1134 observations. They include the following variables: 

–Region: the Municipality Code Number for each of the 54 districts in North Rhine-Westphalia, e.g. “1” for Düsseldorf,

–LifeE: life expectancy in each *region*,

–Year: calendar year from 1990 to 2010, recoded as 1 to 21 for this analysis,

–UrbanRural: indicator, whether a *region* is rural (=0) or urban (=1).

Life expectancy is an important demographic indicator which is computed on basis of the life-table technique. A birth cohort is followed over time and, on the basis of the number of persons that died in every life year, mortality rates are determined which allow the computation of life expectancy. Life expectancy can be calculated conditional upon having reached any given age though it is typically considered from birth as done here. To provide timely life expectancy the current force of mortality (here for NRW) is applied to a hypothetical cohort and provides the data used in this study. Life expectancy computed in this way has to be interpreted as the expected life time for a newborn for the period in which the life table used was computed. For more details on life table techniques see Hinde [7].

The Table 1 shows the names of the regions in association with the numbering used in this analysis. Note that sometimes identical names occur such as Aachen (16) and Aachen (20). The explanation is that these correspond to different areas: the first refers to the city area whereas the second to the ensconcing rural vicinity.

**Table 1:** **Explanation of the variable *region***

**Number**	**Name**	**Number**	**Name**
1	Düsseldorf	28	Bottrop
2	Duisburg	29	Gelsenkirchen
3	Essen	30	Münster
4	Krefeld	31	Borken
5	Mönchengladbach	32	Coesfeld
6	Mülheim a.d. Ruhr	33	Recklinghausen
7	Oberhausen	34	Steinfurt
8	Remscheid	35	Warendorf
9	Solingen	36	Bielefeld
10	Wuppertal	37	Gütersloh
11	Kleve	38	Herford
12	Mettmann	39	Höxter
13	Neuss	40	Lippe
14	Viersen	41	Minden-Lübbecke
15	Wesel	42	Paderborn
16	Aachen (city)	43	Bochum
17	Bonn	44	Dortmund
18	Köln	45	Hagen
19	Leverkusen	46	Hamm
20	Aachen (rural)	47	Herne
21	Düren	48	Ennepe-Ruhr-Kreis
22	Erftkreis	49	Hochsauerlandkreis
23	Euskirchen	50	Märkischer Kreis
24	Heinsberg	51	Olpe
25	Oberbergischer Kreis	52	Siegen-Wittgenstein
26	Rheinisch-Bergischer Kreis	53	Soest
27	Rhein-Sieg-Kreis	54	Unna

Life expectancy is linearly growing in all regions in NRW as Figure 1 indicates. But growth occurs on different levels where the level depends on the area under consideration (see again Figure 1). To explore these regional differences further, a likelihood-based cluster analysis is suggested in the following.

## Methods

### Model and associated likelihoods

We assume that the life expectancy *Y*_*it*_ in region *i* and year *t* is available for *i* = 1, ⋯, *n* and *t* = 1, ⋯, *T*. Note that we use the index *t* starting from 1 instead of the Christian calendar. We further assume that a (latent) component structure is present within the population of regions which has not been observed directly and that within a component the life expectancy in time follows a simple straight model for *j* = 1, ⋯, *J*

(1) $$Y_{\text{it}}=\alpha_{j}+\mathrm{\beta t}+\epsilon_{\text{it}},\text{for}\;\;t=1,\cdots \;,T$$

and that within this component *j* and region *i* the data follow a multivariate normal distribution with diagonal covariance matrix and a common element describing this diagonal

(2) $$\begin{array}{l} f(y_{i}|\alpha_{j},\beta ,\sigma^{2})=\underset{t}{\prod }\phi (y_{\text{it}}|\alpha_{j},\beta ,\sigma^{2})\\ \;=\underset{t}{\prod }\frac{1}{\sqrt{2\Pi \sigma^{2}}}\exp\left(-\frac{1}{2\sigma^{2}}{\left(y_{\text{it}}-\alpha_{j}-\mathrm{\beta t}\right)}^{2}\right), \end{array}$$

where *y*_*i*_ = (*y*_*i*1_, …, *y*_*iT*_)^*T*^ is the *T*-vector of observations of life expectancy in area *i*. Note that this model allows straight lines with potentially *J* different levels. Also note that *σ*^2^ is the variance parameter of the meanzero normal random error *ϵ*_*it*_.

We should point out that we assume here that repeated observations of life expectancy are independent for the 21 observation years *conditional upon component membership j*. This assumption is crucial but not untypical for random effects modelling (see also McLachlan and Peel [6]). We mention that covariance structures could be modelled leading to a multivariate normal distribution for *y*_*i*_ (and ultimately to mixtures of multivariate normals). However, we prefer to remain in the spirit of random effects modelling where we assume that covariance structures are coped with by the introduction of random effects.

Since we do not observe component membership we only take the marginal distribution as a nonparametric mixture

(3) $$\begin{array}{c} \overset{J}{\underset{j=1}{\sum }}f(y_{i}|\alpha_{j},\beta ,\sigma^{2})p_{j}=\overset{J}{\underset{j=1}{\sum }}\underset{t}{\prod }\phi (y_{\text{it}}|\alpha_{j},\beta ,\sigma^{2})p_{j}, \end{array}$$

where the *p*_*j*_ represents the unknown weights of the components in the population.

Consequently, the observable mixture model likelihood is

(4) $$\begin{array}{c} \ell =\underset{i}{\prod }\overset{J}{\underset{j=1}{\sum }}\underset{t}{\prod }\phi (y_{\text{it}}|\alpha_{j},\beta ,\sigma^{2})p_{j}, \end{array}$$

which needs to be maximized in *α*_*j*_, *p*_*j*_ for *j* = 1, ⋯, *J* and *β* and *σ*^2^. Note the special form of the likelihood in its hierarchical structure. Conditional upon component membership it assumes *independence* in time.

Note that this form of random effects modelling is not uncommon for this situation (Arminger *et al.*[8]; Goldstein [9]; Aitkin [10], Ng *et al.*[11], Ram and Grimm [12], Muthén and Asparouhov [13]; Rabe–Hesketh and Skrondal [14]). The central idea is that the random effect copes with the temporal and/or the spatial structure of the data.

Since the observed likelihood function is difficult to maximize in the parameters we consider the unobserved likelihood typical for mixture problems of this kind. Let *z*_*ij*_ denote the unobserved indicator informing about component membership. In other word, *z*_*ij*_ = 1 if the *i*-th region belongs to component *j* and 0 otherwise. Then the unobserved complete likelihood is

(5) $$\begin{array}{l} L=\underset{i}{\prod }\underset{j}{\prod }{\;\left[\underset{t}{\prod }\phi (y_{\text{it}}|\alpha_{j},\beta ,\sigma^{2})p_{j}\right]}^{z_{\text{ij}}}\\ \;=\underset{i}{\prod }\underset{j}{\prod }{\;\left[\underset{t}{\prod }\phi (y_{\text{it}}|\alpha_{j},\beta ,\sigma^{2})\right]}^{z_{\text{ij}}}\times \underset{i}{\prod }\underset{j}{\prod }p_{j}^{z_{\text{ij}}}, \end{array}$$

showing that the likelihood can now be separately maximized in *α*_*j*_, for *j* = 1, ⋯, *J*, *β*, *σ*^2^ on one hand, and *p*_*j*_ for *j* = 1, ⋯, *J* on the other hand. This is best done with the EM algorithm.

The problem is well-posed in the sense that if *J* is fixed the likelihood is bounded and can be maximized. However, if the likelihood is also maximized with respect to *J* as suggested in the approach by Laird [15] and Lindsay [16,17], then the likelihood becomes unbounded as *σ*^2^ approaches 0 (see also Wang [18]). To overcome this problem we follow Aitkin [10] and keep *J* fixed for determining the maximum likelihood estimates and then stepwise vary *J*. We then select the number of components on the basis of the Bayesian Information Criterion (BIC) to search for the best model.

We use the following definition for the BIC

(6) $$\begin{array}{ll} \text{BIC}=-2\ell +r\;\log(n), & \; \end{array}$$

where *r* is the number of estimated parameters and *n* is the number of regions (here *n* = 54). Models are considered suitable with small BIC-value. Another criterion could be

(7) $$\begin{array}{ll} \text{BI}C_{2}=-2\ell +r\;\log(\text{nT}), & \; \end{array}$$

where *T* is the considered number of years (here *T* = 21). Note that *N* = *nT* so that two different penalty terms are possible, namely log(*n*) and log(*nT*), respectively. Given the choice of modelling which considers each area as clustered in time, we find (6) the more appropriate selection criterion which uses the number of areas *n* in the penalty term. This also seems to correspond to common practice (Muthén and Asparouhov [13]). For completeness, we shall compute both.

### Expectation-maximization (EM) algorithm

To estimate the parameters by maximum likelihood we will use the EM algorithm (Dempster, Laird, and Rubin [4]; McLachlan and Krishnan [5]; McLachlan and Peel [6]). The EM algorithm consists of two steps: the *E-Step* and the *M-Step*. The algorithm cycles between these two steps back and forth.

#### E-step

In the E-step the unobserved indicator variables *z*_*ij*_ are replaced by their expected values conditional upon the current parameter estimates and the data *Y*_*it*_

$$e_{\text{ij}}=E(Z_{\text{ij}}|\alpha_{j},p_{j},\beta ,\sigma^{2}).$$

These expected values can be easily computed using Bayes theorem as

$$e_{\text{ij}}=\frac{f_{\text{ij}}p_{j}}{\overset{J}{\underset{k=1}{\sum }}f_{\text{ik}}p_{k}}$$

and can be interpreted as the posterior probability that region *i* belongs to component *j* (note *e*_*ij*_ ≥ 0 and $\sum_{j}e_{\text{ij}}=1$). Here

$$f_{\text{ij}}=\underset{t}{\prod }\phi (y_{\text{it}}|\alpha_{j},\beta ,\sigma^{2}).$$

### M-step

It is easy to see that the likelihood (5) is maximized for *p*_*j*_ as

$${\hat{p}}_{j}=\overset{n}{\underset{i=1}{\sum }}e_{\text{ij}}/\mathrm{n.}$$

For the remainder we concentrate on

$$\;\log L=\underset{i}{\sum }\underset{j}{\sum }e_{\text{ij}}\underset{t}{\sum }\left[-\frac{1}{2}\log\sigma^{2}-\frac{1}{2\sigma^{2}}{\left(y_{\text{it}}\;-\;\alpha_{j}-\mathrm{\beta t}\right)}^{2}\right].$$

Setting the partial derivative $\frac{\partial }{\partial \alpha_{j}}\log L=0$ leads to

$${\hat{\alpha }}_{j}=\frac{\underset{i}{\sum }e_{\text{ij}}\underset{t}{\sum }(y_{\text{it}}-\beta t)}{T\underset{i}{\sum }e_{\text{ij}}}.$$

Similarly, setting all other partial derivatives to 0 we achieve

$$\hat{\beta }=\frac{\underset{i}{\sum }\underset{j}{\sum }e_{\text{ij}}\underset{t}{\sum }(y_{\text{it}}-\alpha_{j})t}{(\underset{i}{\sum }\underset{j}{\sum }e_{\text{ij}})\underset{t}{\sum }t^{2}}$$

and

$${\hat{\sigma }}^{2}=\frac{\underset{i}{\sum }\underset{j}{\sum }e_{\text{ij}}\underset{t}{\sum }{\left(y_{\text{it}}-\alpha_{j}-\mathrm{\beta t}\right)}^{2}}{T\underset{i}{\sum }\underset{j}{\sum }e_{\text{ij}}}.$$

Here *e*_*ij*_, *p*_*j*_, *α*_*j*_, *β* and *σ*^2^ refer to the values of these parameters in the previous cycle of the EM algorithm.

The EM algorithm toggles between E- and M-step until convergence, say until *Δ* is less than *ϵ* where *ϵ* is a small number such as 0.0001. *Δ* refers to the absolute difference in each of the parameters between two consecutive cycles *s* + 1 and *s*, for example $\Delta =|\alpha_{j}^{s+1}-\alpha_{j}^{s}|$ if we consider the intercept.

### Initial values

We need to compute initial values for the variables *e*_*ij*_, *p*_*j*_, *α*_*j*_, *β* and *σ*^2^. Only for this purpose we fit the following linear model:

(8) $$\begin{array}{ll} Y_{\text{it}}=a_{i}+b_{i}t+\epsilon_{\text{it}},\;t=1,\ldots ,T & \; \end{array}$$

for each region *i* = 1, …, *n*, separately leading to *n* estimates of *a*_*i*_, *b*_*i*_ and $\sigma_{i}^{2}$ denoted as $\hat{a_{i}}$, $\hat{b_{i}}$ and $\hat{\sigma_{i}^{2}}$. Now we use these estimates to get our starting values for the EM algorithm:

$$\begin{array}{lll} \alpha_{j}^{0} & =\text{j-th quantile of}\;\;\hat{a_{i}}\; & \;\\ \beta^{0} & =\text{median}(\hat{b_{i}})\; & \;\\ \sigma^{2^{0}} & =\text{median}(\hat{\sigma_{i}^{2}})\; & \; \end{array}$$

Additionally we initialize $(p_{1}^{0},\ldots ,p_{J}^{0})=\left(\frac{1}{J},\ldots ,\frac{1}{J}\right)$. Then we run the EM algorithm for various values of *J* starting with the homogeneity case *J* = 1 to get estimates of *e*_*ij*_, *p*_*j*_, *α*_*j*_, *β* and *σ*^2^. With these values we compute the (maximized) likelihood (4).

## Results

### Cluster structure

Table 2 shows the results of the EM algorithm for men for *J* = 1, …, 10. As we can see the values for *BIC* decrease with growing index *J* until *J* = 7 just to increase again. The values for *BIC*_2_ show the same behaviour but have the minimum at *J* = 6. We also run the EM algorithm for the female data for *J* = 1, …, 10. Again the *BIC* is decreasing but now we find the minimum at *J* = 8. The optimal *J* lies between 6 and 8 regardless of the different selection criteria. Overall, it seems appropriate to take *J* = 7 which splits the data into 7 different category groups. Table 2 provides also estimates for the slope *β* and the variance *σ*^2^. The choice of *J* = 7 appears also justified when we consider the value of *σ*^2^ in dependence of *J* which becomes stable at *J* = 7. Note that the slope estimate is stable independent of the choice of *J*. Details of the full estimation results of the mixing distribution are provided in Table 3.

**Table 2:** **Model evaluation for men and women, *J *= 1, …, 10**

***J***	***β***		***σ***^**2**^		***BIC***		***BIC***_**2**_	
	**Men**	**Women**	**Men**	**Women**	**Men**	**Women**	**Men**	**Women**
1	0.2560	0.1673	0.8969	0.5147	1503.98	1497.21	1513.12	1506.34
2	0.2560	0.1673	0.4407	0.2593	1499.24	1359.64	1514.46	1374.86
3	0.2560	0.1673	0.3079	0.2088	1455.63	1294.24	1476.94	1315.56
4	0.2560	0.1673	0.2651	0.1882	1434.90	1264.26	1462.30	1291.66
5	0.2560	0.1673	0.2308	0.1760	1396.11	1246.16	1429.60	1279.65
6	0.2560	0.1673	0.2114	0.1655	1369.02	1239.95	**1408.60**	1279.53
7	0.2560	0.1673	0.2045	0.1594	**1364.20**	1232.36	1409.87	**1278.02**
8	0.2560	0.1673	0.2014	0.1566	1365.88	**1229.81**	1417.63	1281.57
9	0.2560	0.1673	0.2014	0.1547	1373.86	1232.48	1431.70	1290.33
10	0.2560	0.1673	0.2012	0.1545	1381.61	1240.11	1445.55	1304.05

**Table 3:** **Estimated cluster structure of life expectancy growth level for men and women, *J *= 7**

***J***	***p***_***j***_		***α***_***j***_	
	**Men**	**Women**	**Men**	**Women**
7	0.0556	0.0185	70.32	77.27
	0.1841	0.1112	71.19	78.08
	0.1529	0.2655	71.76	78.54
	0.2711	0.1978	72.23	78.84
	0.1700	0.1684	72.72	79.29
	0.0922	0.2016	73.07	79.63
	0.0740	0.0371	73.71	80.22

### Maximum posteriori classification

#### Men

Since each *e*_*ij*_ describes the probability that region *i* belongs to component *j*, we can easily identify to which component each region belongs to according to the maximum posterior probability rule (MAP). The MAP classifies region *i* into component *j* where

(9) $$\begin{array}{ll} e_{\text{ij}}=\underset{l}{\max}e_{\text{il}}. & \; \end{array}$$

**Figure 2:**
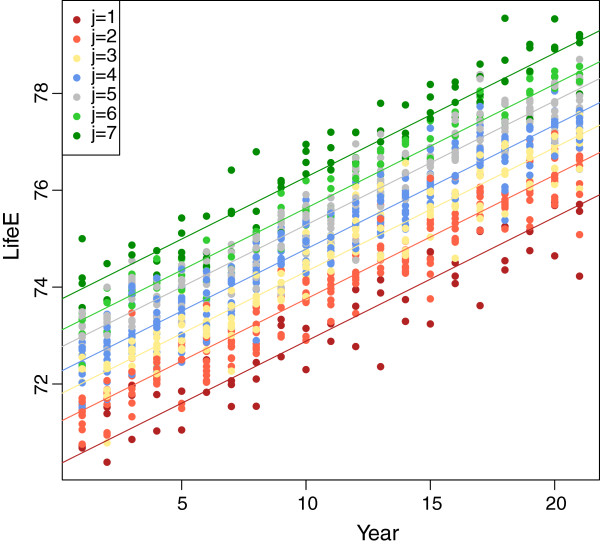
Life expectancy for men, coloured by the component membership.

The classification tables are given in Table 4. This classification is the second column of Table 4 where one can also see the matrix *e*_*ij*_ (rounded to 2 digits after the decimal point). Note that in all cases the classification is unique in the sense that there is a high classification probability for a particular component. Now we are able to construct a graph wherein the datapoints are coloured by the different components where they belong to (Figure 2).

**Table 4:** **Maximum posterior classification (MAP) for men, *J *= 7**

**Region**	**Name**	**Class**	***e***_***.*1**_	***e***_***.*2**_	***e***_***.*3**_	***e***_***.*4**_	***e***_***.*5**_	***e***_***.*6**_	***e***_***.*7**_
1	Düsseldorf	2	0	0.95	0.05	0	0	0	0
2	Duisburg	1	1	0	0	0	0	0	0
3	Essen	2	0	1	0	0	0	0	0
4	Krefeld	4	0	0	0	1	0	0	0
5	Mönchengladbach	2	0	1	0	0	0	0	0
6	Mülheim a.d. Ruhr	4	0	0	0.01	0.99	0	0	0
7	Oberhausen	1	1	0	0	0	0	0	0
8	Remscheid	2	0	1	0	0	0	0	0
9	Solingen	4	0	0	0	1	0	0	0
10	Wuppertal	3	0	0	1	0	0	0	0
11	Kleve	3	0	0	1	0	0	0	0
12	Mettmann	6	0	0	0	0	0.03	0.97	0
13	Neuss	6	0	0	0	0	0.01	0.99	0
14	Viersen	4	0	0	0	1	0	0	0
15	Wesel	4	0	0	0	1	0	0	0
16	Aachen (city)	6	0	0	0	0	0	1	0
17	Bonn	7	0	0	0	0	0	0	1
18	Köln	4	0	0	0.03	0.97	0	0	0
19	Leverkusen	5	0	0	0	0	1	0	0
20	Aachen (rural)	4	0	0	0.16	0.84	0	0	0
21	Düren	4	0	0	0	1	0	0	0
22	Erftkreis	5	0	0	0	0	1	0	0
23	Euskirchen	3	0	0	1	0	0	0	0
24	Heinsberg	4	0	0	0	1	0	0	0
25	Oberbergischer Kreis	4	0	0	0	1	0	0	0
26	Rheinisch-Bergischer Kreis	7	0	0	0	0	0	0	1
27	Rhein-Sieg-Kreis	7	0	0	0	0	0	0	1
28	Bottrop	2	0	1	0	0	0	0	0
29	Gelsenkirchen	1	1	0	0	0	0	0	0
30	Münster	7	0	0	0	0	0	0	1
31	Borken	4	0	0	0	0.96	0.04	0	0
32	Coesfeld	6	0	0	0	0	0.14	0.86	0
33	Recklinghausen	2	0	1	0	0	0	0	0
34	Steinfurt	5	0	0	0	0	1	0	0
35	Warendorf	5	0	0	0	0	0.96	0.04	0
36	Bielefeld	5	0	0	0	0	1	0	0
37	Gütersloh	6	0	0	0	0	0	1	0
38	Herford	5	0	0	0	0	1	0	0
39	Höxter	5	0	0	0	0	0.99	0.01	0
40	Lippe	5	0	0	0	0	0.92	0.08	0
41	Minden-Lübbecke	4	0	0	0.01	0.99	0	0	0
42	Paderborn	5	0	0	0	0	1	0	0
43	Bochum	2	0	1	0	0	0	0	0
44	Dortmund	2	0	1	0	0	0	0	0
45	Hagen	2	0	1	0	0	0	0	0
46	Hamm	3	0	0	1	0	0	0	0
47	Herne	2	0	1	0	0	0	0	0
48	Ennepe-Ruhr-Kreis	3	0	0	1	0	0	0	0
49	Hochsauerlandkreis	4	0	0	0	0.88	0.12	0	0
50	Märkischer Kreis	3	0	0	1	0	0	0	0
51	Olpe	4	0	0	0	1	0	0	0
52	Siegen-Wittgenstein	4	0	0	0	1	0	0	0
53	Soest	3	0	0	1	0	0	0	0
54	Unna	3	0	0	0.99	0.01	0	0	0

In addition to the data points we have included in Figure 2 the regression lines for each component with the parameters from Table 2.

#### Women

**Figure 3:**
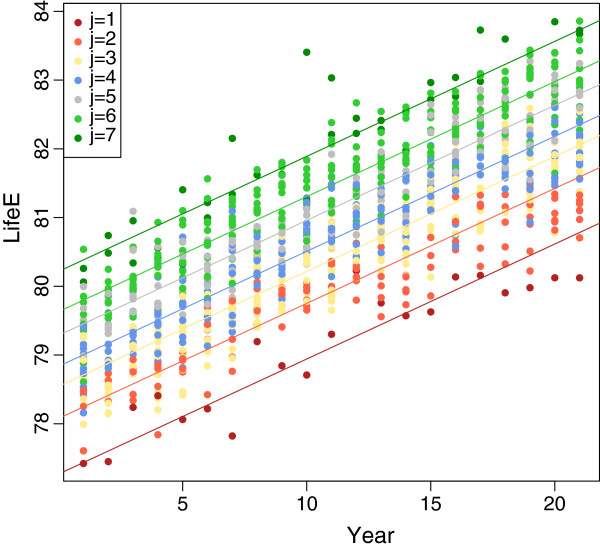
Life expectancy for women, coloured by component membership.

**Figure 4:**
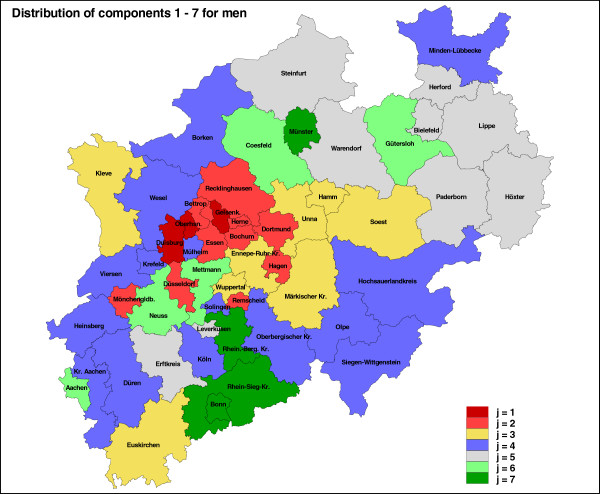
Geographical map of the classification of the 54 regions of NRW into the 7 components of life expectancy, 1 (red) low to 7 (green) high, men.

In Table 5 we consider women. It again consists of the *Region*, the corresponding component for each region and the entire matrix *e*_*ij*_ for *J* = 7. With this information we construct the plot of life expectancy for women whereas the data points are colored by the different components (Figure 3). Note that the associated maps of the estimated cluster/component structure of the different levels of life–expectancy are provided as Figures 4 (for men) and 5 (for women).

**Table 5:** **Maximum posterior classification (MAP) for women, *J *= 7**

**Region**	**Name**	**Class**	***e***_***.*1**_	***e***_***.*2**_	***e***_***.*3**_	***e***_***.*4**_	***e***_***.*5**_	***e***_***.*6**_	***e***_***.*7**_
1	Düsseldorf	3	0	0	0.98	0.02	0	0	0
2	Duisburg	2	0	1	0	0	0	0	0
3	Essen	3	0	0.05	0.95	0	0	0	0
4	Krefeld	4	0	0	0.01	0.99	0	0	0
5	Mönchengladbach	2	0	1	0	0	0	0	0
6	Mülheim a.d. Ruhr	4	0	0	0	1	0	0	0
7	Oberhausen	2	0	1	0	0	0	0	0
8	Remscheid	3	0	0	1	0	0	0	0
9	Solingen	4	0	0	0	0.96	0.04	0	0
10	Wuppertal	4	0	0	0.12	0.88	0	0	0
11	Kleve	3	0	0	1	0	0	0	0
12	Mettmann	5	0	0	0	0	1	0	0
13	Neuss	5	0	0	0	0	1	0	0
14	Viersen	3	0	0	0.98	0.02	0	0	0
15	Wesel	4	0	0	0.13	0.87	0	0	0
16	Aachen (city)	5	0	0	0	0	1	0	0
17	Bonn	7	0	0	0	0	0	0	1
18	Köln	3	0	0	0.88	0.12	0	0	0
19	Leverkusen	5	0	0	0	0	1	0	0
20	Aachen (rural)	3	0	0	1	0	0	0	0
21	Düren	3	0	0	0.97	0.03	0	0	0
22	Erftkreis	3	0	0	0.83	0.17	0	0	0
23	Euskirchen	3	0	0	1	0	0	0	0
24	Heinsberg	3	0	0	0.79	0.21	0	0	0
25	Oberbergischer Kreis	4	0	0	0	1	0	0	0
26	Rheinisch-Bergischer Kreis	6	0	0	0	0	0	1	0
27	Rhein-Sieg-Kreis	5	0	0	0	0	0.74	0.26	0
28	Bottrop	3	0	0.01	0.99	0	0	0	0
29	Gelsenkirchen	1	1	0	0	0	0	0	0
30	Münster	7	0	0	0	0	0	0	1
31	Borken	5	0	0	0	0	0.98	0.02	0
32	Coesfeld	6	0	0	0	0	0	1	0
33	Recklinghausen	2	0	0.94	0.06	0	0	0	0
34	Steinfurt	6	0	0	0	0	0	1	0
35	Warendorf	6	0	0	0	0	0.44	0.56	0
36	Bielefeld	6	0	0	0	0	0	1	0
37	Gütersloh	6	0	0	0	0	0	1	0
38	Herford	6	0	0	0	0	0	1	0
39	Höxter	6	0	0	0	0	0.09	0.91	0
40	Lippe	6	0	0	0	0	0	1	0
41	Minden-Lübbecke	6	0	0	0	0	0.01	0.99	0
42	Paderborn	6	0	0	0	0	0.02	0.98	0
43	Bochum	3	0	0	1	0	0	0	0
44	Dortmund	2	0	1	0	0	0	0	0
45	Hagen	4	0	0	0	1	0	0	0
46	Hamm	4	0	0	0.27	0.73	0	0	0
47	Herne	2	0	1	0	0	0	0	0
48	Ennepe-Ruhr-Kreis	4	0	0	0	1	0	0	0
49	Hochsauerlandkreis	5	0	0	0	0	1	0	0
50	Märkischer Kreis	3	0	0	1	0	0	0	0
51	Olpe	5	0	0	0	0	0.84	0.16	0
52	Siegen-Wittgenstein	5	0	0	0	0.06	0.94	0	0
53	Soest	4	0	0	0.32	0.68	0	0	0
54	Unna	4	0	0	0.07	0.93	0	0	0

**Figure 5:**
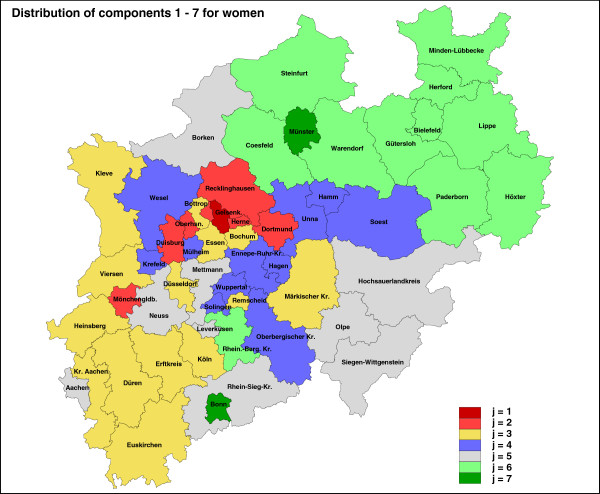
Geographical map of the classification of the 54 regions of NRW into the 7 components of life expectancy, 1 (red) low to 7 (green) high, women.

### Explaining the cluster structure

The data contain also a variable characterizing each area as rural (= 0) or urban (= 1). The results can be summarized into the following cross-classified tables (Table 6, Table 7).

**Table 6:** Cluster classification (men)

	**1**	**2**	**3**	**4**	**5**	**6**	**7**	∑
Rural	0	1	6	11	7	4	2	31
Urban	3	9	2	4	2	1	2	23
∑	3	10	8	15	9	5	4	54

**Table 7:** Cluster classification (women)

	**1**	**2**	**3**	**4**	**5**	**6**	**7**	∑
Rural	0	1	8	5	7	10	0	31
Urban	1	5	6	6	2	1	2	23
∑	1	6	14	11	9	11	2	54

A simple chi-square test investigates the relation between these two categorical variables: classification using the performed cluster analysis and the binary variable rural/urban. We find for men: *χ*^2^ = 18.4645 by 6 DF and p-value = 0.00517, which is highly significant. For women we find: *χ*^2^ = 15.3361 by 6 DF and p-value = 0.00178, clearly significant.

**Figure 6:**
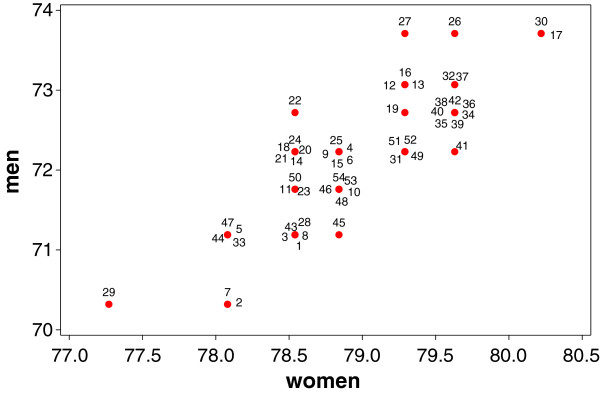
**Scatterplot of life-expectancy level **$\alpha_{j(i)}^{w}$**for women against the life-expectancy level**$\alpha_{j(i)}^{m}$**for men of region *i*, *j(i) *is the component *j *into which the region *i *is classified.**

We conclude the results section with a final analysis as follows. We have done a separate cluster analysis for men and women. For men, a particular region will be classified into a component, but for women this region might be classified into a different component. To provide a consistent analysis both classifications should be correlated. This is what the last graphic is about. Figure 6 shows the connection between the components for each region for men and women. There we can identify for which regions the life expectancy for both men and women is high or low. For example, region 30 (Münster) and 17 (Bonn) are for men and women in the highest level of life expectancy growth, whereas region 29 (Gelsenkirchen) is in lowest for both gender groups.

## Discussion and conclusion

The normal density (2) is frequently used as mixture kernel and appropriate for our application. However, if necessary it allows easy extensions either in the mean structure or the variance-covariance structure. For one, one could allow component–specific variances leading to $f(y_{i}|\alpha_{j},\beta ,\sigma_{j}^{2})$. In addition, one could also think of giving up independence within area *i* leading to a multivariate normal distribution with either structured or completely unstructured variance–covariance matrix *Σ*. Furthermore, one could think of modelling component–specific variance–covariance matrices *Σ*_*j*_. For two, instead of using a common slope model this could be generalized to component-specific slopes *β*_*j*_ leading *f*(*y*_*i*_|*α*_*j*_, *β*_*j*_, *σ*^2^) or $f(y_{i}|\alpha_{j},\beta_{j},\sigma_{j}^{2})$. The E-step of the EM algorithm has to be changed appropriately, in the case of a common variance parameter *σ*^2^ leading to

$${\hat{\alpha }}_{j}=\frac{\underset{i}{\sum }e_{\text{ij}}\underset{t}{\sum }(y_{\text{it}}-\beta t)}{T\underset{i}{\sum }e_{\text{ij}}},$$

as before, and

$${\hat{\beta }}_{j}=\frac{\underset{i}{\sum }e_{\text{ij}}\underset{t}{\sum }(y_{\text{it}}-\alpha_{j})t}{(\underset{i}{\sum }e_{\text{ij}})\underset{t}{\sum }t^{2}}$$

which reflects the fact that there are component–specific slopes and

$${\hat{\sigma }}^{2}=\frac{\underset{i}{\sum }\underset{j}{\sum }e_{\text{ij}}\underset{t}{\sum }{\left(y_{\text{it}}-\alpha_{j}-\beta t\right)}^{2}}{T\underset{i}{\sum }\underset{j}{\sum }e_{\text{ij}}}.$$

However, for our data constellation the proposed model (2) is appropriate as also Figure 1 suggests. A more rigorous analysis for this assumption requires fitting the model with random intercepts only and also the model allowing the slopes to be random as well. This has be done using a mixed model approach with a normal random effects assumption. The BIC-values associated with the model fits support the random-intercept only assumption.

Also, we have looked at the potential for curvature. This would correspond to an asymptotic change in life expectancy growth and relates to the discussion in Oeppen and Vaupel [1]. For males, the log-likelihood for the model with a quadratic term for year is -2*ℓ* = 2919.0, whereas the log-likelihood for the model without the quadratic terms is -2*ℓ* = 2920.2, leading to a likelihood ratio test statistic value of 1.2 with p-value 0.27, clearly not significant. For females, we have similar results.

A qualitatively different approach follows a conditional autoregressive model (CAR) which was originally suggested by Clayton and Kaldor [19] and more recently modified by Rasmussen [20]. In principle, the idea could be also utilized for spatial-temporal modelling as in this case and tries to implement a smoothing element by utilizing spatial information. The key element of CAR models is to model mean and covariance structure of the random effect (here the intercept in the temporal straight line model) by neighboring information. The ultimate goal is to reach a smooth map of the measure of interest (here level of life expectancy growth). This approach is quite meaningful, in particular, if the underlying process is likely to have a smooth characteristic. In our case, however, we were more interested in identifying a potential clustered structure in life expectancy growth for which we thought the likelihood based cluster approach is more appropriate. Hence we have not followed up on CAR models in this case.

In North Rhine-Westphalia (NRW), there is an apparent continuous rise in life expectancy at birth in men and women within the last twenty years. However, this pattern needs to be contemplated differentially. Our analysis shows that in North Rhine-Westphalia, life expectancy is predominantly higher in rural than in urban districts and differs considerably by region. Within the observed period from 1990 to 2010, levels of growths of life expectancy ranged from 70.3 to 73.7 years in men and from 77.3 to 80.2 in women. Life expectancy in the 54 districts was influenced by a latent categorical variable, which consists of seven categories or clusters. Each of the 54 districts is allocated into one of the seven clusters. This latent variable might be a surrogate variable for socio-economic factors. Life expectancy, as well as its counterpart mortality, strongly depends on factors like education, income, occupational status in addition to the factors sex and age. Most recent analyses of the European Prospective Investigation into Cancer and Nutrition (EPIC) showed that total mortality among men with highest education level is reduced by 43% compared to men with the lowest (hazard ratio (HR): 0.57, 95% confidence interval (CI) 0.52 – 0.61). Among women, the reduction was 29% (HR 0.71, 95% CI 0.64 – 0.78). In men, social inequalities were highly statistically significant for all causes of death examined. In women, the authors found a less strong, but statistically significant association with social inequalities for all causes of death except for cancer mortality and injuries (Gallo *et al.*[3]). For the region 29 (Gelsenkirchen), we found the lowest life expectancy for both, men and women. Socio-economic factors (see also *Health reporting unit at the NRW Centre for Health*[21]) support this finding and point to possible underlying causes of this result. For Gelsenkirchen, the lowest disposable income per inhabitant in NRW is documented (2009: 15,905 Euros / inhabitant; 80.8% of NRW average) as well as the highest rate of persons drawing unemployment benefits (12,189.7/100,000 inhabitants in 2009). In Gelsenkirchen, we observed the highest death rate per 100,000 inhabitants in 2010: 1,338.7 (Standardized Mortality Ratio (SMR): 1.17; NRW in total: 1.00). In 2009, only in Herne (47) and Dortmund (44) the proportion of smokers was higher (Gelsenkirchen: 31.4%; Herne: 35.0%; Dortmund: 32.3%). The opposite extreme of longest life expectancy for both sexes was found for two cities. Newborn girls and boys can expect the longest life in the regions 17 (Bonn) and 30 (Münster). In 2010, for Bonn and Münster the lowest SMRs of all NRW districts were documented (SMR: Bonn 0.83 / Münster 0.87). In contrast to Gelsenkirchen these cities have the lowest rate of persons receiving unemployment benefits (Bonn: 5,738.5/100,000; Münster: 5,090.3/100,000). Large universities are based in Bonn and Münster with thousands of students as city inhabitants. Therefore, the disposable income per inhabitant is above NRW average, but other regions show higher income rates. The proportion of smokers is relatively low in both cities (Bonn: 22.9%; Münster: 23.7%). In 2009, only in five rural districts the proportion of smokers was lower. Results for men and women differ slightly, as was reported for social inequalities in the EPIC cohort, too (Gallo *et al.*[3]). In men, besides Gelsenkirchen the cities Duisburg (2) and Oberhausen (7) are classified as regions with the lowest life expectancy of NRW. In women, it is only Gelsenkirchen.

These findings support results of a socio-spatial cluster analysis conducted in 2007 by Strohmeier *et al.*[2] which was mentioned already in the introduction. Based on social indicators six clusters were established for NRW, which classified the 54 districts into six types which were dubbed as follows: poverty pole, family zone, cities dominated by administrative and service units, rising regions / suburban counties, heterogeneous cities, heterogeneous rural districts. Like in our analysis, the poverty pole (representing areas which are in several ways socially deprived) included the cities Gelsenkirchen, Duisburg, and Oberhausen, but also the cities Dortmund and Herne which are all located in the Ruhr area.

In relation to the NRW health indicators the authors found a significantly lower male and female average life expectancy in the poverty pole. In our analyses also more cities, especially of the Ruhr area, are categorized into the clusters of lower life expectancy. The Ruhr area is an urbanized, high density area comprising 11 cities and 4 counties with about 5 million inhabitants, formerly characterized by heavy industry and now undergoing a structural change towards e.g. information technology and health care industry. An additional underlying cause for lower life expectancy in this area might still be environmental. The Heinz Nixdorf RECALL study (Fuks *et al.*[22]), which included 4,291 participants from the Ruhr cities Bochum (43), Essen (3) and Mülheim a.d. Ruhr (6), recently confirmed that residential proximity to high road traffic (≤ 50m) and road traffic noise exposure (24h mean noise (Lden) > 65 dB) have a tendency toward higher blood pressure and an elevated prevalence of hypertension. Data from this study also showed that a reduction in distance between residence and major roads by half was associated with a 7.0% (95% CI 0.1 – 14.4%) higher coronary artery calcification (CAC) (Hoffmann *et al.*[23]).

In a subgroup of the RECALL study population, participants residing in Essen (n=1,641) and Mülheim (n=1,742) for which digitized information on inner city roads was available, prevalence of coronary heart disease at high traffic exposure showed significantly elevated OR=1.85 (95% CI 1.21 – 2.84, adjusted for cardiovascular risk factors and background air pollution) (Hoffmann *et al.*[24]). Further analysis showed a stronger effect for men (OR=2.33, 95% CI 1.44 – 3.78), which might account for the difference among men and women in our cluster analysis. Another analysis of the RECALL data investigated if the association of road traffic exposure and subclinical cardiovascular disease might be modified by socio-economic characteristics of individuals or neighborhoods. Participants with low socio-economic status (SES) and simultaneous exposure to high road traffic had highest levels of CAC (Dragano *et al.*[25]). The prevalence of high CAC was 23.9% in higher-educated men with low traffic exposure but 37.7% in lower-educated men with high road traffic exposure (women: 22.0% vs. 28.1%).

The cluster analysis of life expectancy once more stresses the differences between urban and rural regions in North Rhine-Westphalia. The latent component categorizing the 54 districts into seven categories can be interpreted as a surrogate comprising several underlying factors. The results point to districts where an accumulation of problems has negative impact on health. For males, only three cities are classified into the lowest cluster category, with 5.4% of the total NRW population living there. For women, only Gelsenkirchen is classified into this cluster. Given the emerging insight into possible underlying causes, chances for these cities to improve their outcome may come into closer reach.

## Competing interests

The authors declare that they have no competing interests.

## Authors’ contributions

All authors have made substantial individual contributions to the manuscript. All authors read and approved the final manuscript.

## Authors’ information

Claudia Terschüren, Rolf Annuß and Rainer Fehr are public health scientists working for the Landeszentrum Gesundheit NRW, Germany. Sarah Karasek is a postgraduate student of statistics at the University of Graz and Dankmar Böhning is Chair in Medical Statistics at the University of Southampton.

## Pre-publication history

The pre-publication history for this paper can be accessed here:

http://www.biomedcentral.com/1471-2288/13/36/prepub
